# Loss of Metabotropic Glutamate Receptor 5 Function on Peripheral Benzodiazepine Receptor in Mice Prenatally Exposed to LPS

**DOI:** 10.1371/journal.pone.0142093

**Published:** 2015-11-04

**Authors:** Dany Arsenault, Katherine Coulombe, Aijun Zhu, Chunyu Gong, Kun-Eek Kil, Ji-Kyung Choi, Pekka Poutiainen, Anna-Liisa Brownell

**Affiliations:** 1 Athinoula A. Martinos Center for Biomedical Imaging, Department of Radiology, Massachusetts General Hospital, Harvard Medical School, Charlestown, Massachusetts, United States of America; 2 Centre de Recherche du CHU de Québec, Axe Neurosciences, Faculté de Pharmacie, Université Laval, Québec, QC, Canada; University of Minnesota, UNITED STATES

## Abstract

Parental microglial induced neuroinflammation, triggered by bacterial- or viral infections, can induce neuropsychiatric disorders like schizophrenia and autism to offspring in animal models. Recent investigations suggest that microglia, the resident immune cells of the brain, provides a link between neurotransmission, immune cell activation, brain inflammation and neuronal dysfunction seen with the offspring. Relatively little is known about how reduction of brain inflammation and restoration of glial function are associated with diminution of brain degeneration and behavioral deficits in offspring. Increased mGluR5 expression and the long-lasting excitotoxic effects of the neurotoxin during brain development are associated with the glial dysfunctions. We investigated the relationship of mGluR5 and PBR and how they regulate glial function and inflammatory processes in mice prenatally exposed to LPS (120μg/kg, between gestational days 15 and 17), an inflammatory model of a psychiatric disorder. Using PET imaging, we showed that pharmacological activation of mGluR5 during 5 weeks reduced expression of classic inflammation marker PBR in many brain areas and that this molecular association was not present in LPS-exposed offspring. The post-mortem analysis revealed that the down regulation of PBR was mediated through activation of mGluR5 in astrocytes. In addition, we demonstrated that this interaction is defective in a mouse model of the psychiatric deficit offering a novel insight of mGluR5 involvement to brain related disorders and PBR related imaging studies. In conclusion, mGluR5 driven glutamatergic activity regulates astrocytic functions associated with PBR (cholesterol transport, neurosteroidogenesis, glial phenotype) during maturation and could be associated with neuropsychiatric disorders in offspring.

## Introduction

In recent years, metabotropic glutamate receptor subtype 5 (mGluR5) has been a growing topic in research for its role in several central and peripheral diseases [1]. Its signaling is mainly associated to G_q_/G_11_ and activates phospholipase C, resulting in hydrolysis of phosphoinositides and generation of inositol 1,4,5-triphosphate and diacylglycerol. This typical pathway leads to calcium mobilization and activation of protein kinase C [2]. The mGluR5 is found on postsynaptic terminals of neurons and in glial cells [3]. The molecular pathways associated to neuronal mGluR5 and its therapeutic potential in different pathologies such as schizophrenia, anxiety and Parkinson’s disease are largely reviewed in the literature [4–8].

The expression of mGluR5 profusely increases in glial cells activated by inflammatory stimulus [9, 10]. The glial mGluR5 activation is known to play a role in gliotransmission triggering the intercellular communication between neurones and glial cells [11]. Pathophysiological impairments of glial mGluR5 are associated with the development of behavioral disorders [12–15]. The underlying mechanism is related to decreased glutamate reuptake and suppression of mGluR5 dependent synaptic plasticity leading to enhanced astroglial loss. Transient up-regulation of mGluR5 in microglia and astrocyte was observed in different neurodevelopmental and neuroinflammatory models and associated with behavioral abnormalities in adulthood [12–15]. The activation of mGluR5 reduces the amount of reactive glial cells, brain inflammation and neurotoxicity [10, 12, 16, 17]. These anti-inflammatory properties are associated with a lower level of brain degeneration and a reduction of behavioral deficits. This led to hypothesis of the possible underlying interaction between mGluR5, inflammation and glial function.

Peripheral benzodiazepine receptor (PBR, also called translocator protein (TSPO) 18 kDa) is a potent target for mGluR5 to modulate glial function. PBR is a small protein primarily localized in the outer mitochondrial membrane of glial cells (microglia and astrocytes) [18]. It plays a key role in the transport of cholesterol into mitochondria and in neurosteroidogenesis [19–21]. Recently, PBR has received attention as a potent inflammatory marker in many animal models of brain pathology [7, 22–25], since it is expressed in glial cells activated by an inflammatory process. Moreover, pharmacological activation of PBR decreases the inflammation in glial cells [26]. The preclinical studies have promoted the development of PBR markers to image brain inflammation in humans and animals by positron emission tomography (PET) [27]. In humans, imaging studies targeting PBR have reported a specific increase in the regions affected in neurodegenerative diseases such as Alzheimer’s [28, 29] and Parkinson’s disease [30, 31], and in other neurological pathologies like ischemic stroke [32] and multiple sclerosis [33, 34]. These studies have supported the idea that PBR is a sensor of brain injury or defect, and the recovery of the brain function can be quantitatively detected by PET imaging [18, 35].

The aim of this study was to investigate the effect of mGluR5 activity on PBR expression and astrocyte activation in the mice prenatally exposed to LPS. The LPS was administered into the mother (dose of 120 μg/Kg i.p.) [36] to induce brain inflammation in the fetus [37]. The pharmacological effect of mGluR5 modulation was evaluated using adult offspring treated during 5 weeks with mGluR5 agonist (CDPPB, 10mg/kg) or antagonist (MTEP, 3mg/kg). Both of these drugs are known to cross the blood brain barrier [38, 39] and the used doses are known to be effective at a behavioral level [5, 40–43]. Finally, the obtained results of mGluR5 and glial activation in the brain were verified with *ex vivo* analyses.

## Materials and Methods

### Animals

Altogether 30 pregnant C57BL/6 mice were purchased from Charles River Laboratories (Wilmington, Massachusetts), handled in our in-house breeding facility and kept in ventilated cages under standard laboratory conditions. Experimental studies were conducted in 92 pups. The animal studies were approved by the Subcommittee on Research Animals of Massachusetts General Hospital and the Harvard Medical School and carried out by the Guide of the National Institute of Health for the Care and Use of Laboratory Animals.

### Prenatal immune challenges

**Fig 1:**
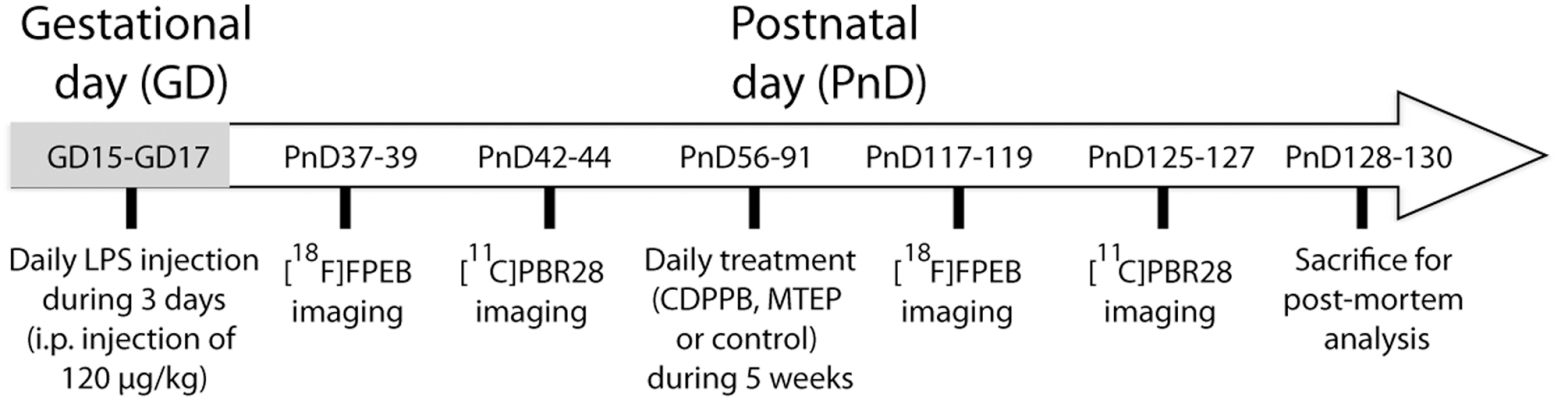
Time lines of the experiments. Pregnant mice received an injection of 120 μg/kg of LPS or an equivalent volume of saline between GD15 to GD17. Expression of mGluR5 (PnD37-PnD39) and PBR (PnD42-PnD44) in the brain of the offspring prenatally exposed to LPS or saline were evaluated by PET imaging. Animals of both groups (LPS or saline) were then exposed to treatment during 5 weeks (from PnD56 to PnD91). Three types of treatments were tested: CDPPB (10 mg/kg; mGluR5 agonist), MTEP (3 mg/kg; mGluR5 antagonist) and control (solvent alone). Finally, the *in vivo* expression of mGluR5 (PnD117-119) and PBR (PnD125-127) were re-evaluated after the treatments and the animals were sacrificed a few days after the last imaging session (PnD128-130) for post-mortem analysis. Abbreviations: GD, gestational day; [^11^C]PBR28, peripheral benzodiazepine receptor 28; [^18^F]FPEB, [^18^F]fluoro-5-(2-pyridinylethynyl)benzonitrile; PnD, postnatal day.>>

Bacterial infection was induced in pregnant mice by one intraperitoneal (i.p.) injection of LPS (*E*.*coli* serotype 0111:134, 120 μg/kg/day; 0.05 ml/g; Sigma-Aldrich, Missouri, USA). Equivalent volume (0.05 ml/g) of sterile saline solution was used for the control treatment. Treatments were administered during the late stage of gestation, i.e. between GD15 and GD17 (Fig 1). Note that doses and delivery modes were chosen based on the current literature according to the known impact in inducing neuroinflammation in foetal brain after one injection within the same time window as in our studies [13, 37]. This prenatal immune challenge model was chosen for three reasons; first, it produced a transient upregulation of mGluR5 expression during development [13], confirming a physiological alteration of this receptor by the inflammatory processes; second, pups prenatally exposed to this treatment showed a delay in the development of sensorimotor reflex [13]; and third, psychiatric symptoms (anxiety impairments and pre-pulse inhibition deficit) in adult offspring were reported in rodents prenatally exposed to LPS in late pregnancy [14, 37, 44, 45]. The two last points confirm that this prenatal immune challenge interfere sufficiently with the development process to induce behavioral changes later.

### Drug treatment

Mice received a daily intraperitoneal injection (at 9 am) from the postnatal day 56 (PnD56) to PnD91 during 5 weeks, see Fig 1. Negative allosteric modulator of mGluR5, 3-((2-methyl-4-thiazolyl)ethynyl)pyridine (MTEP), (2 mg/ml, 3 mg/kg animal, Tocris, Cat. No. 2921) and positive allosteric modulator of mGluR5, 3-Cyano-N-(1,3-diphenyl-1H-pyrazol-5-yl) benzamide (CDPPB), (6.66 mg/ml, 10 mg/kg animal, Tocris, Cat. No. 3235) solutions were prepared with the same dissolving solution (accordingly to manufacturer’s recommendation). Drug preparation was made so that each animal received equivalent volume (1.5 μl/g animal). Equivalent volume of dissolving solution was used for the control treatment. Fresh solutions were prepared every 2–3 days and preserved at 4°C. The CDPPB and MTEP doses used in this study are known to be active at the behavioral and cellular level [5, 40–43]. Since mGluR5 is known to play a function during the cerebral development and in the adult brain, the treatment was started in adulthood to exclude the effect of mGluR5 on development and, consequently, limiting the present results to the effect of mGluR5 in mature/adult brain.

### PET Ligands and Imaging

PET imaging included studies of mGluR5 using [^18^F]FPEB ([^18^F]fluoro-5-(2-pyridinylethynyl)benzonitrile) and studies of inflammatory response using [^11^C]PBR28 ([^11^C]peripheral benzodiazepine receptor 28) as radiolabeled ligands. For the imaging studies, animals were anesthetized with isoflurane/oxygen (1–1.5% isoflurane at 1 L/min oxygen flow). Catheterization of tail vein was done for the administration of the radiolabeled ligands. The animal was adjusted into the scanner (Triumph II, Trifoil Imaging Inc). The level of anesthesia and vital signs were monitored throughout the imaging with the monitoring system of the scanner. For the PET imaging studies, radiolabeled ligand ([^18^F]FPEB or [^11^C]PBR28, 0.2 mCi, specific activity of 1900 mCi/μmol for [^18^F]FPEB and 600–1000 mCi/μmol for [^11^C]PBR28) was injected into the tail vein and dynamic volumetric data were acquired for 60 minutes. After the PET data acquisition, computed tomography (CT) imaging was done to obtain data for attenuation correction and anatomical information.

PET data were reconstructed with an algorithm based on maximum likelihood estimation using 30 iterations. CT data was reconstructed with a modified Feldkamp algorithm using matrix volumes of 512×512×512 and pixel size of 170 μm. The regions of interest including striatum, frontal cortex, hippocampus, hypothalamus, cerebellum, olfactory bulb and whole brain were drawn on all coronal and axial levels using co-registered axial, sagittal, and coronal CT-PET images of the brain. Activity per unit volume, percent activity of the injected dose and the ligand concentration were calculated.

Kinetic analysis to determine binding potential of [^18^F]FPEB was done using PMOD 3.208 software (PMOD Technologies LTD, Zurich, Switzerland) and reference tissue method with the cerebellum data as an input function. Since the expression of mGluR5 in cerebellum is minimal [46] and the radioactivity emitted by the [^18^F]FPEB ligand is similar between cerebellum and blood after 60 minutes [47], the input function can be processed from the cerebellum data in calculating regional maps for binding potential. This approach is more reliable than using only a percent of the injected activity per cm^3^ since the background is removed. Concerning [^11^C]PBR28, cerebellum cannot be used as a reference tissue, since inflammation is not brain area specific. The binding for [^11^C]PBR28 was determined as a, percent of the injected activity per cm^3^. Binding of the ligand to the receptor is an indication of the activity of the receptor since the ligand only binds to membranous receptors.

The effect of different postnatal treatments on the binding values of [^18^F]FPEB and [^11^C]PBR was determined by comparing to the binding values obtained after the control treatment. Each binding value in different brain areas and different treatments was divided by the average value obtained of the same brain structure of mice, which received the same prenatal treatment but a control solution as postnatal treatment (solvent without active compound). Since postnatal treatment is the changing factor in this ratio, the value what is significantly different from the value of one confirms the effect of the treatment. A significant result over the value of one means that the postnatal treatment increases the binding potential of the quantified receptor, whereas if the value is significantly below the value of one, the treatment decreases the binding potential. This normalization confirms only the variations induced by the postnatal treatments (agonist or antagonist of mGluR5) and simplifies data presentation.

### Tissue preparation

Perfusion was done under deep anesthesia with ketamine (Vetalar, Bioniche, Ontario, Canada) and xylazine (Bayer, Ontario, Canada) (i.p. injection of 10 and 0.1 mg/kg, respectively). Brains were extracted and snap frozen on dry ice. The entire right hemisphere was used for western blot analyses.

### Protein extraction and western immunoblotting

For *post mortem* analyses, 8 volumes of lysis buffer (150 mM NaCl, 10 mM NaH2PO4, 1% Triton X-100, 0.5% SDS, and 0.5% deoxycholate) containing CompleteTM protease inhibitor cocktail (Roche, Indianapolis, USA), 10 mg/ml of pepstatin A, 0.1 mM EDTA and phosphatase inhibitors (1 mM each of sodium vanadate and sodium pyrophosphate, 50 mM sodium fluoride, Sigma-Aldrich, Missouri, USA) were added to frozen brain samples (entire right hemisphere), sonicated (3 x 10 sec), centrifuged at 100,000 *g* for 20 min at 4°C and the supernatant was collected.

The protein concentration in the supernatants was determined using a BCA protein assay kit (Pierce, Illinois, USA) according to manufacturer’s protocol. Equal amounts of protein per sample were diluted in Laemmli’s loading buffer. The samples were then heated for 5 min at 95°C, before loading to a SDS-PAGE gel. Proteins were transferred onto PVDF membranes (Millipore, Massachusetts, USA), before blocking in 5% non-fat dry milk and 1% bovine serum albumin (BSA) in PBS-0.1% Tween20 for 1 hour. Membranes were immunoblotted with appropriate primary and secondary antibodies followed by chemiluminescence reagents (Lumiglo Reserve, KPL, Maryland, USA). Band intensities were quantified using a KODAK Image Station 4000 MM Digital Imaging System (Molecular Imaging Software version 4.0.5f7, Carestream Health, New York, USA). For this study the following antibodies were used: goat polyclonal anti-Iba1 (ionized calcium binding adaptor molecule-1, cat. number: NB100-2833, 1:500, Novus Biological), mouse monoclonal anti-GFAP (glial fibrillary acidic protein, cat. number: G3893, 1:10000, Sigma-Aldrich), rabbit polyclonal anti-mGluR5 (cat. number: AB5675, 1:1000, Millipore), rabbit anti-CD68 (cluster of differentiation 68, cat. number: 16192-1-AP, 1:500, Proteintech) and mouse monoclonal anti-actin (cat. number: G043, 1:5000, abm). Note that specific denaturating conditions were necessary to dissociate the dimerization of mGluR5 [48, 49]. Consequently, the dimeric form of mGluR5 was quantified, as previously reported and published [13, 48].

### Statistical analyses

**Fig 2:**
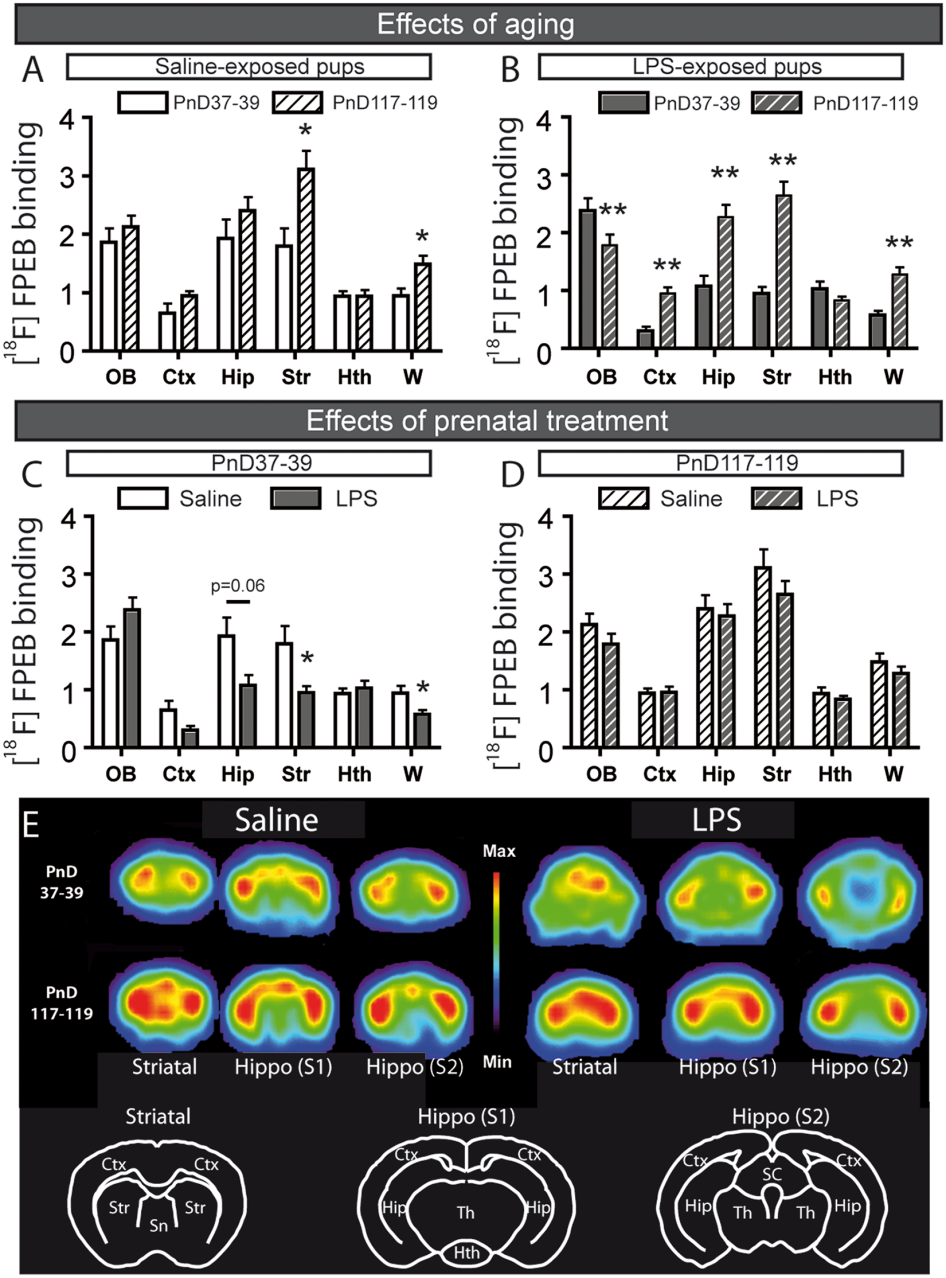
Effect of aging and different prenatal treatments on mGluR5 expression investigated with PET imaging using [^18^F]FPEB. PET imaging revealed a significant increase of [^18^F]FPEB binding during maturation (from PnD37-39 to PnD 117–119) in the striatum and the whole brain of the mice prenatally exposed to saline (A), while in the LPS-exposed offspring the [^18^F]FPEB binding increased in the cortex, hippocampus, striatum and the whole brain during the same maturation period (B). During the adolescence (PnD37-39) we observed a lower binding of [^18^F]FPEB in the striatum and the whole brain of the LPS-exposed offspring compared to the saline-treated offspring (C). However, [^18^F]FPEB binding was similar in both groups at PnD117-119 (D). (E) Coronal slices of hippocampal and striatal level of [^18^F]FPEB in offspring prenatally exposed to saline or LPS at PnD 37–39 and PnD 117–119. Values are expressed as mean ± SEM. Abbreviations: [^18^F]fluoro-5-(2-pyridinylethynyl)benzonitrile; Ctx, cortex; Hip, hippocampus; Hth, hypothalamus; OB, olfactory bulb; PnD, postnatal day; Str, striatum; W, whole brain. *p < 0.05, **p < 0.01 >>

**Fig 3:**
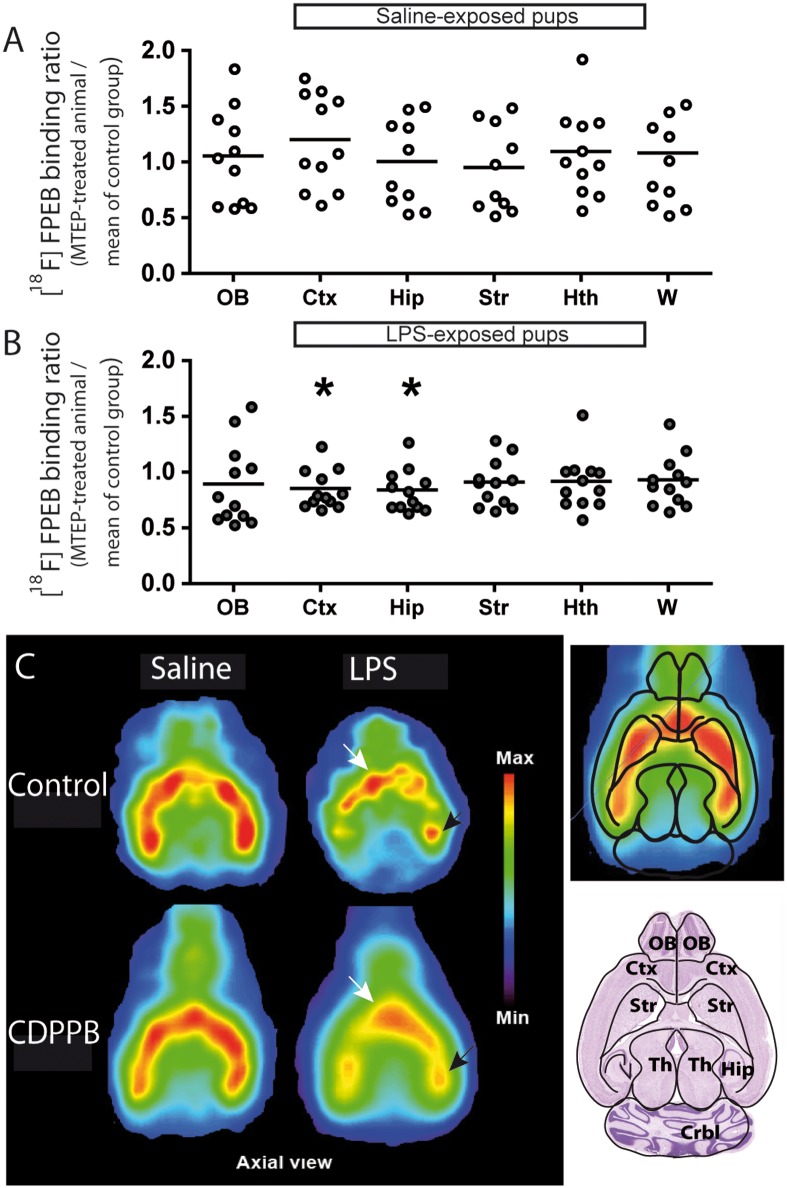
Effects of postnatal CDPPB treatment on [^18^F]FPEB binding potential. CDPPB treatment did not change the [^18^F]FPEB binding potential in the quantified brain region of the offspring prenatally exposed to saline solution (A). However, the LPS-exposed offspring had a lower [^18^F]FPEB accumulation in the cortex and hippocampus following CDPPB treatment (B). (C) Axial view of a representative mouse from each group. Values are expressed as mean ± SEM. Abbreviations: CDPPB, 3-cyano-*N*-(1,3-diphenyl-1H-pyrazol-5-yl)benzamide; [^18^F]FPEB, [18F]fluoro-5-(2-pyridinylethynyl)benzonitrile; Ctx, cortex; Hip, hippocampus; Hth, hypothalamus; OB, olfactory bulb; PnD, postnatal day; Str, striatum; W, whole brain. *p < 0.05, **p < 0.01. Statistical analyses were performed using one-sample t test (all comparisons of panel A; OB, Hip, Str, Hth and W of panel B) or Wilcoxon signed-rank test (Ctx of panel B). The number of animals was 10–11 for the saline group and 12 for the LPS-exposed group. >>

**Fig 4:**
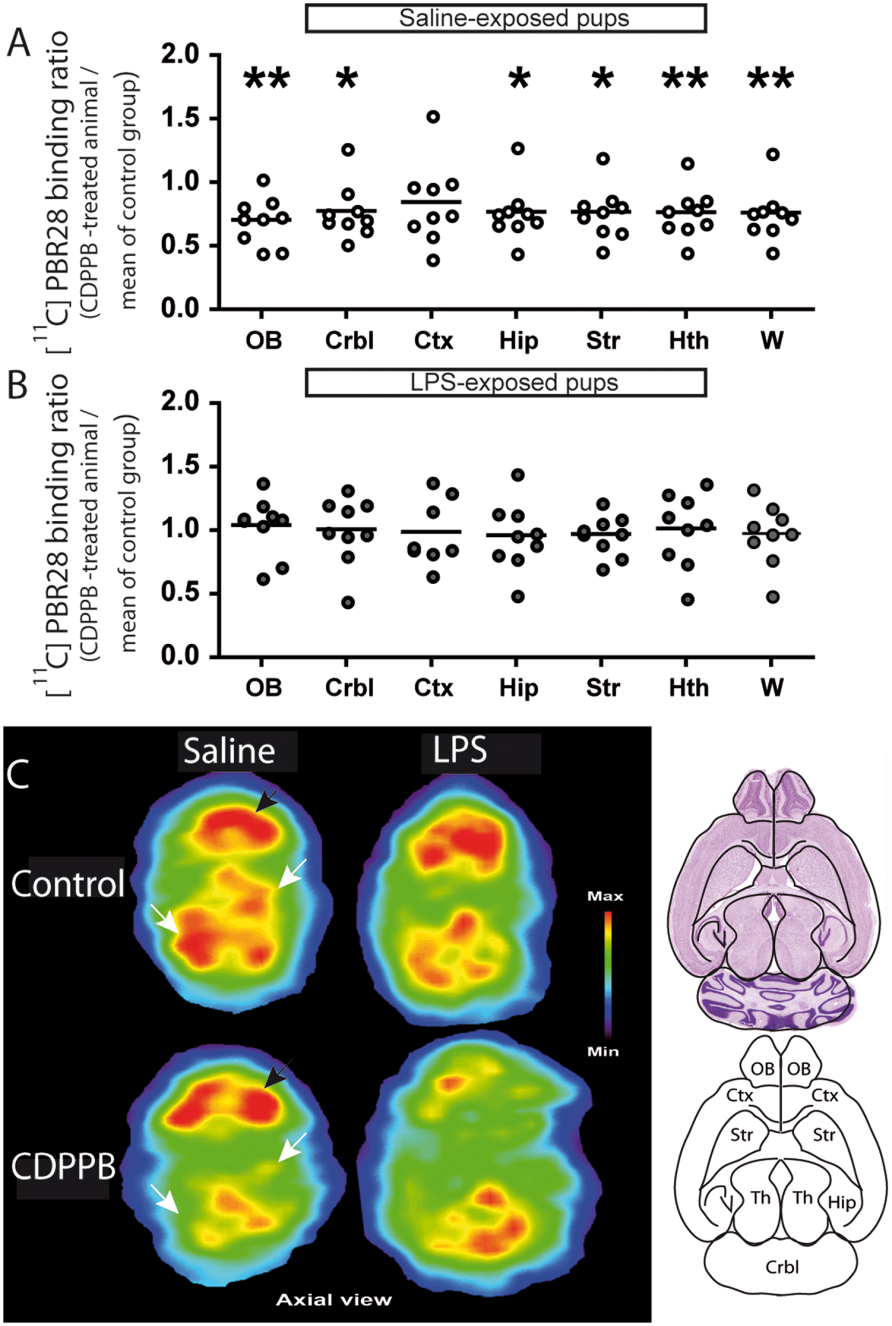
Postnatal CDPPB treatment decreased [^11^C]PBR accumulation in saline-exposed offspring, but not in offspring prenatally exposed to LPS. Postnatal CDPPB treatment decreased the accumulation of [^11^C]PBR in the olfactory bulb, cerebellum, hippocampus, striatum, hypothalamus and the whole brain of the offspring prenatally exposed to saline solution (A). However, [^11^C]PBR accumulation did not change in any quantified brain region of the LPS-exposed offspring (B). Axial PET images of [^11^C]PBR accumulation at the midbrain level illustrate the decreased accumulation after CDPPB treatment in the prenatally saline-exposed offspring while there is no significant change in the brain of CDPPB treated mice prenatally exposed to LPS (C). Values are expressed as mean ± SEM. Abbreviations: CDPPB, 3-cyano-*N*-(1,3-diphenyl-1*H*-pyrazol-5-yl)benzamide; [^11^C] PBR28, peripheral benzodiazepine receptor 28; Ctx, cortex; Crbl, cerebellum; Hip, hippocampus; Hth, hypothalamus; OB, olfactory bulb; PnD, postnatal day; Str, striatum; W, whole brain. *p < 0.05, **p < 0.01 >>

**Fig 5:**
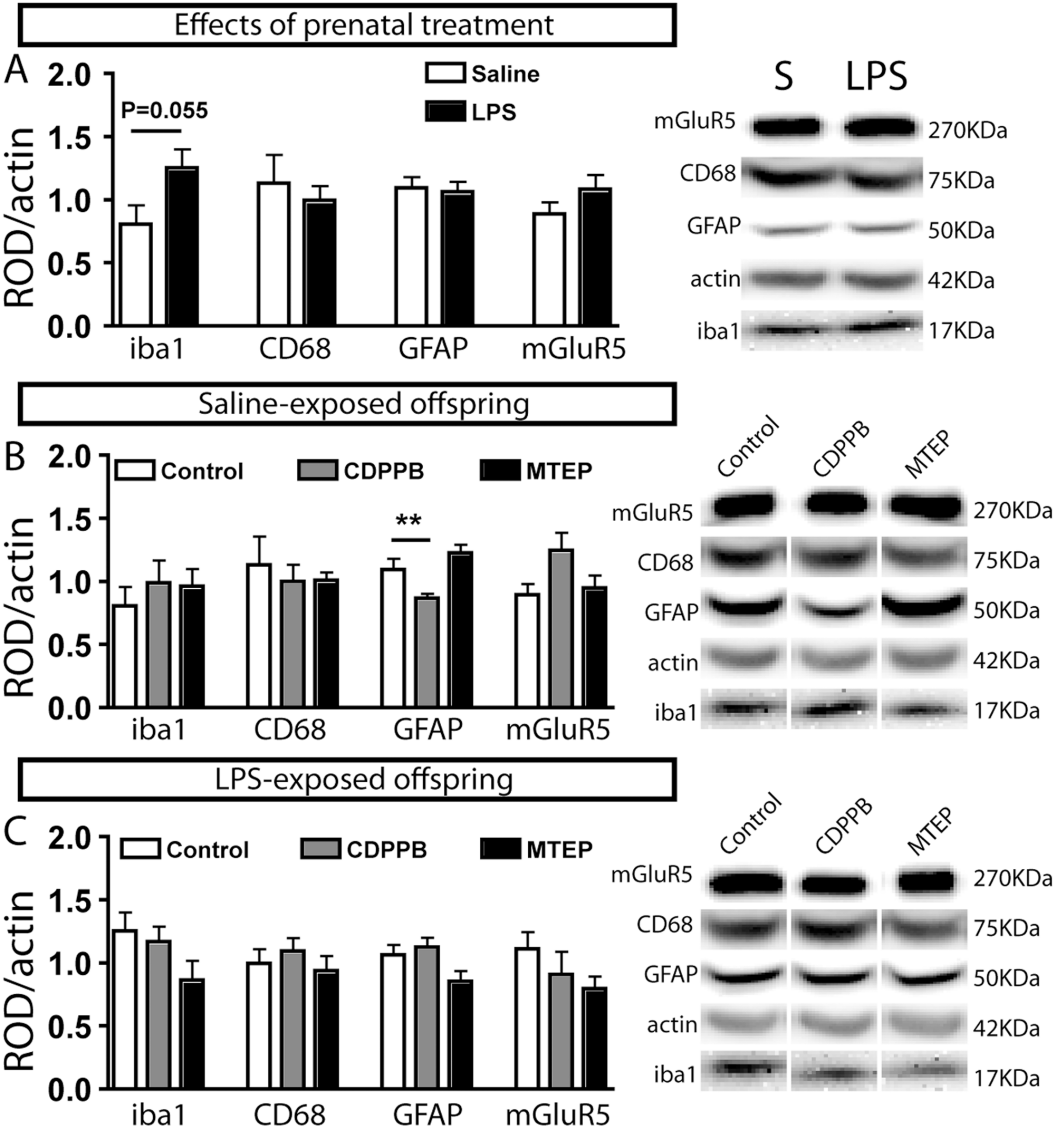
Astrocytic marker was decreased in the brain of prenatally saline-exposed offspring, but not in the offspring prenatally exposed to LPS, following pharmacological activation of mGluR5. A prenatal administration of LPS did not change the brain expression level of iba1, CD68, GFAP or mGluR5 in 4-month-old mice (A). Postnatal CDPPB treatment reduced the GFAP level, without effect on iba1, CD68 or mGluR5 in the mice prenatally exposed to saline solution. The quantified molecular markers did not change in the brain of mice treated with MTEP (B). No change in iba1, CD68, GFAP or mGluR5 was observed in the brain of the mice prenatally exposed to LPS (C). Values are expressed as mean ± SEM. Abbreviations: MTEP, 3-((2-methyl-4-thiazolyl)ethynyl)pyridine; CDPPB, 3-cyano-*N*-(1,3-diphenyl-1H-pyrazol-5-yl) benzamide; CD68, cluster of differentiation 68); iba1, ionized calcium binding adaptor molecule-1; GFAP, glial fibrillary acidic protein; mGluR5, metabotropic glutamate receptor subtype 5; ROD, relative optical density. **p < 0.01 >>

Statistical comparisons between groups were performed according to normality of distribution and variance equivalences between the groups. In cases of equal variance and normal distribution we used an unpaired Student’s t-test to compare 2 groups (Fig 2, S1 Fig). Nonparametric Mann-Whitney test was used to compare groups in which the distribution was not confirmed. When the variance was unequal, groups were compared using a Welch’s correction. For matched values, paired t-test (parametric) or Wilcoxon matched pairs test (nonparametric) was performed. When a parameter was normalized to the relevant population, a one-sample t test (parametric) or Wilcoxon signed-rank test (nonparametric) was used. The effects of postnatal treatment were analyzed by dividing each value with the average of the group receiving the same prenatal treatment (Figs 3 and 4, S2 and S3 Figs). A value significantly different of one meant that the treated group was different from the control group. We used also an analysis of variance (ANOVA) followed by Dunnett post hoc test to compare each treatment with a control/reference group (Fig 5B and 5C). Finally, the effect of gender was verified for each comparison and both sexes were analyzed separately, only when the difference was observed. The statistics were not protected by multiple comparisons. All statistical analyses were performed using JMP (version 9, SAS) and prism (version 4.0, GraphPad Software Inc.).

## Results

### [^18^F]FPEB binding potential was altered during maturation of LPS-exposed offspring

During maturity, we observed an increase of [^18^F]FPEB binding potential in the striatum and the whole brain of saline-exposed offspring (Fig 2A and 2E). Statistical analyses (Table 1) were performed using paired t-test (olfactory bulb (OB), cortex (Ctx), hippocampus (Hip), striatum (Str) and whole brain (W)) or Wilcoxon matched pairs test (hypothalamus (Hth)).

**Table 1:** Statistical values. The statistical methods used and obtained p-values in each treatment group and brain region.

	Statistics	U, T, F or W value / p value	Statistics	U, T, F or W value / p value
**Fig 2. (FPEB quantification)**				
Brain	**panel A**		**panel B**	
region	*Saline-exposed offspring (PnD37-39 vs PnD117-119)*		*LPS-exposed offspring (PnD37-39 vs PnD117-119)*	
OB	Paired T-test	T (8) = 0.872 / p = 0.409	Paired T-test	T (7) = 4.729 / **p = 0.002**
Ctx	Paired T-test	T (8) = 1.755 / p = 0.117	Paired T-test	T (6) = 4.155 / **p = 0.006**
Hip	Paired T-test	T (9) = 1.183 / p = 0.267	Paired T-test	T (6) = 3.984 / **p = 0.007**
Str	Paired T-test	T (8) = 2.848 / **p = 0.022**	Paired T-test	T (6) = 5.823 / **p = 0.001**
Hth	Paired T-test	T (9) = 0.064 / p = 0.951	Paired T-test	T (7) = 1.498 / p = 0.118
W	Paired T-test	T (8) = 2.629 / **p = 0.030**	Paired T-test	T (7) = 4.044 / **p = 0.005**
Brain	**panel C**		**panel D**	
region	*PnD37-39 (saline vs LPS prenatal exposition)*		*PnD117-119 (saline vs LPS prenatal exposition)*	
OB	Unpaired T-test	T (15) = 1.562 / p = 0.139	Unpaired T-test	T (23) = 1.234 / p = 0.230
Ctx	Welch's correction	T (11) = 1.856 / p = 0.090	Mann-Whitney Test	U (180, 145) = 75.00 / p = 0.935
Hip	Unpaired T-test	T (15) = 2.001 / p = 0.064	Unpaired T-test	T (23) = 0.396 / p = 0.696
Str	Welch's correction	T (10) = 2.500 / **p = 0.031**	Mann-Whitney Test	U (198, 127) = 61.00 / p = 0.396
Hth	Mann-Whitney Test	U (100, 71) = 35.00 / p = 0.697	Welch's correction	T (20) = 0.765 / p = 0.453
W	Unpaired T-test	T (15) = 2.251 / **p = 0.040**	Mann-Whitney Test	U (188, 112) = 57.00 / p = 0.464
**Fig 3. (FPEB binding potential)**				
Brain	**panel A**		**panel B**	
region	*Effect of CDPPB in saline-exposed offspring (PnD117-119)*		*Effect of CDPPB in LPS-exposed offspring (PnD117-119)*	
OB	One sample t test	T (10) = 0.437 / p = 0.671	One sample t test	T (11) = 1.015 / p = 0.332
Crbl	———	———	———	———
Ctx	One sample t test	T (10) = 1.580 / p = 0.145	One sample t test	T (11) = 2.901 / **p = 0.014**
Hip	One sample t test	T (9) = 0.049 / p = 0.962	One sample t test	T (11) = 2.888 / **p = 0.015**
Str	One sample t test	T (9) = 0.398 / p = 0.700	One sample t test	T (11) = 1.496 / p = 0.163
Hth	One sample t test	T (10) = 0.813 / p = 0.435	One sample t test	T (11) = 1.193 / p = 0.258
W	One sample t test	T (10) = 0.566 / p = 0.584	One sample t test	T (11) = 1.037 / p = 0.322
**Fig 4. (PBR quantification)**				
Brain	**panel A**		**panel B**	
region	*Effect of CDPPB in saline-exposed offspring (PnD125-127)*		*Effect of CDPPB in LPS-exposed offspring (PnD117-119)*	
OB	One sample t test	T (8) = 4.679 / **p = 0.002**	Wilcoxon signed rank	W (30.00, -15.00) = 15.00 / p = 0.426
Crbl	One sample t test	T (8) = 3.114 / **p = 0.014**	One sample t test	T (8) = 0.111 / p = 0.915
Ctx	One sample t test	T (8) = 1.438 / p = 0.188	Wilcoxon signed rank	W (18.00, -18.00) = 0 / p = 1.000
Hip	One sample t test	T (8) = 3.126 / **p = 0.014**	One sample t test	T (8) = 0.437 / p = 0.673
Str	One sample t test	T (8) = 3.352 / **p = 0.010**	One sample t test	T (8) = 0.566 / p = 0.587
Hth	One sample t test	T (8) = 3.605 / **p = 0.007**	One sample t test	T (8) = 0.144 / p = 0.889
W	Wilcoxon signed rank	W (5.00, -40.00) = -35.00 **p = 0.039**	One sample t test	T (8) = 0.275 / p = 0.790
**Fig 5. (Brain protein level)**				
Brain	**panel A**		**panel B**	
protein	*Effect of prenatal treatment in brain protein level*		*Effect of postnatal treatment in saline-exposed offspring*	
mGluR5	Mann Whitney test	U (56,80) = 20.00 / p = 0.235	one-way ANOVA	F (2, 20) = 2.769 / p = 0.087
CD68	One sample t test	T (14) = 0.531 / p = 0.604	one-way ANOVA	F (2, 21) = 0.217 / p = 0.807
iba1	One sample t test	T (13) = 2.123 / p = 0.055	one-way ANOVA	F (2, 20) = 0.364 / p = 0.754
GFAP	One sample t test	T (14) = 0.247 / p = 0.808	one-way ANOVA	F (2, 19) = 6.841 / **p = 0.006**
Brain	**panel C**			
protein	*Effect of postnatal treatment in LPS-exposed offspring*			
mGluR5	one-way ANOVA	F (2, 20) = 1.150 / p = 0.337		
CD68	one-way ANOVA	F (2, 21) = 0.501 / p = 0.613		
iba1	one-way ANOVA	F (2, 20) = 2.119 / p = 0.727		
GFAP	one-way ANOVA	F (2, 21) = 3.073 / p = 0.068		

In mice prenatally exposed to LPS, the binding potential of [^18^F]FPEB decreased in the OB during maturation, but increased in the Ctx, Hip, Str and W (Fig 2B and 2E). Statistical analyses were done using paired t-test (OB, Hip and Hth) or Wilcoxon matched pairs test (Ctx, Str and W). Prenatal immune challenge significantly reduced the binding potential of [^18^F]FPEB in the striatum and the whole brain of adolescent mice (PnD37) compared to the control mice (prenatally saline-exposed offspring, Fig 2C and 2E), while no difference was observed between the adult mice (PnD119; Fig 2D and 2E). Unpaired Student’s t-test (OB, Hip and W of panel C; OB and Hip of panel D), Welch t-test (Ctx and Str of panel C; Hth of panel D) and Mann-Whitney test (Hth of panel C; Ctx, Str and W of panel D) were used to compare the effects of the prenatal treatments. The number of animals was 9–10 (PnD37-39) and 14 (PnD117-119) for the saline-exposed offspring, whereas it was 7–8 (PnD37-39) and 10–11 (PnD117-119) for the LPS-exposed group.

#### Aging and prenatal treatment did not influence [^11^C]PBR28 accumulation

Quantification of [^11^C]PBR28 accumulation showed a significant increase during maturation in the cerebellum of saline-exposed mice (S1A and S1E Fig) without significant change in mice prenatally exposed to LPS (S1B and S1E Fig). No difference of [^11^C]PBR28 accumulation was observed between mice prenatally exposed to saline or LPS at PnD35 (S1C and S1E Fig) and PnD120 (S1D and S1E Fig).

#### Effects of postnatal MTEP treatment on [^18^F]FPEB binding potential

MTEP treatment reduced [^18^F]FPEB binding potential in hypothalamus of saline-treated mice at PnD119 (S2A Fig), but had no effect on mice prenatally exposed to LPS (S2B Fig).

#### [^11^C]PBR28 accumulation was not changed after MTEP treatment

The binding potential of [^11^C]PBR28 in different brain structures was not changed by MTEP treatment in mice prenatally exposed to saline or LPS (S3A and S3B Fig).

#### Effects of postnatal CDPPB treatment on [^18^F]FPEB binding potential

Postnatal treatment with CDPPB did not change the binding potential of [^18^F]FPEB in prenatally saline-exposed offspring (Fig 3A and 3C). However, the binding potential of [^18^F]FPEB decreased in the cortex and hippocampus of LPS-exposed offspring after 5-week CDPPB treatment (Fig 3B and 3C).

#### [^11^C]PBR28 accumulation was decreased in saline-exposed offspring after postnatal CDPPB treatment, but not in offspring prenatally exposed to LPS

CDPPB treatment decreased the binding potential of [^11^C]PBR28 in olfactory bulb, cerebellum, hippocampus, striatum and hypothalamus of saline-exposed offspring (Fig 4A and 4C), but had no effect on LPS-exposed offspring (Fig 4B and 4C). Statistical analyses were performed using one-sample t test (all comparisons of panel A; Crbl, Hip, Str, Hth and W of panel B) or Wilcoxon signed-rank test (OB and Ctx of panel B). The number of animals was 9 for the saline group and 8–9 for the LPS group.

### Effects of prenatal and postnatal treatments on mGluR5 expression and markers of activated glial cells in the brain

Prenatal exposure of LPS did not change mGluR5, iba1, CD68 or GFAP expression in the homogenized whole brain of 128/130-day old animals (Fig 5A). Postnatal CDPPB treatment reduced GFAP expression in saline-exposed offspring, without any change in iba1, CD68 or mGluR5 expression (Fig 5B). No molecular change was observed following MTEP treatment in animals prenatally exposed to saline solution (Fig 5B). Neither treatments (CDPPB or MTEP) changed iba1, CD68, GFAP or mGluR5 expression in the homogenized whole brain of LPS-exposed offspring (Fig 5C). Statistical analyses were performed using unpaired t-test (panel A) or one way ANOVA followed by Dunnett post hoc tests (panel B and C). The number of samples was 7–8 for each group.

## Discussion

### During maturation [^18^F]FPEB binding modulated in many brain structures of offspring prenatally exposed to LPS

Several epidemiological [50–52] and preclinical studies [45, 52, 53] have provided strong evidence that prenatal infections significantly increase the risk for various brain-related developmental disorders [54], including several neurological and neuropsychiatric diseases. We have recently demonstrated that inflammatory processes induced by an infection during gestational period alters expression of glial mGluR5, and the degree of this alteration is associated with many brain-related disorders as a delay in the reflex development of young pups [13], deficits in social behavior and working memory of adolescent offspring [12] and hypoanxious phenotype in young adults [14]. These studies support an idea that modulation of glial mGluR5 expression during development is one factor involved in the brain-related behavioral disorders which are associated with inflammatory processes during development.

In this study, we demonstrated the similar [^18^F]FPEB binding in the adults which were either prenatally exposed or not exposed to LPS, suggesting that prenatal treatment did not influence mGluR5 expression in the adult mice. [^18^F]FPEB binding potential between adolescent and adult mice prenatally exposed to saline solution was similar, proposing that mGluR5 expression is stable at PnD37. In the contrary, prenatally LPS-exposed adolescent mice showed a lower level of [^18^F]FPEB binding than adult mice proposing an instability or incomplete maturation in the expression of mGluR5 at the age of PnD37. The reason of this decrease is uncertain, since mGluR5 is expressed in glial cells and in neurons [3] and many other physiological parameters effect on mGluR5 expression during development (splice variant expression, mRNA, protein modifications and regional expression) [55–57]. Our data supports the idea that mGluR5 could be a target in the treatment of brain-related developmental disorders and to diagnose neurodevelopmental abnormalities by imaging approaches.

#### mGluR5 activity effects on the level of PBR and GFAP in the brain

Our study clearly demonstrated that mGluR5 activation induced by mGluR5 agonist, CDPPB decreased PBR level, a marker of activated glial cells [7, 18] in many brain structures (Figs 4A and 5). This is the first demonstration to show an interaction between these two proteins. To investigate cell type involved in the downregulation of brain PBR, we have quantified by western blot different molecular markers, associated specifically with microglia or astrocyte in the whole brain of animals. GFAP is expressed in astrocytes and its expression is increased in astroglial response [58, 59] whereas the level of iba1 and CD68, two microglial proteins, increases in activated microglia [60]. We have observed a lower level of GFAP following CDPPB treatment, without any change in microglial markers. These results suggested that mGluR5 activity down-regulates PBR level in astrocytes. Supporting active role of mGluR5 in astrocytes, D’ascenzo et al. showed that glutamatergic synaptic function activated mGluR5-dependent astrocytic Ca2+ oscillations and gliotransmission in the nucleus accumbens of mice [11]. The anti-inflammatory action of PBR in astrocytes was previously demonstrated in a rat model of neuropathic pain [61]. In this model, authors demonstrated that pharmacological activation of PBR in a rat model of neuropathic pain prevented GFAP overexpression, reduced astroglial response and decreased the release of TNF-α, without change in microglial markers [61]. In addition, they demonstrated the key role of PBR-dependent synthesis of neurosteroid in the regulation of GFAP expression and astrocyte phenotype [61]. All the data supports the idea that the regulation of PBR by mGluR5, as demonstrated in this study, is a key metabolic pathway by which glutamatergic synaptic activity regulates astrocytic functions.

#### Absence of mGluR5 functional connection effects on PBR and GFAP markers in offspring prenatally exposed to LPS

We have observed that mGluR5 agonist treatment did not change the level of GFAP and PBR in the offspring prenatally exposed to LPS, demonstrating that the functional connection of mGluR5 is lost in this prenatal immune challenge model (Fig 5). We have previously reported in the same model a transient loss of NeuN (neuronal specific nuclear protein), mGluR5 and GFAP (astrocytic marker) in the brain of the foetus exposed to LPS, whereas the brain level of mGluR5 and TNF-α increased [13]. The level of these three markers returned to baseline level at PnD10 [13]. One hypothesis to explain the uncoupling link between mGluR5 and GFAP/PBR may be that the end of gestation can be a key period for the development of inter-cellular link between astrocytes and neurons. The downregulation of both neuron and astrocyte markers in foetal brain following maternal exposition to LPS in late gestation supports this later [13]. The recoveries of these two cell-type markers at PnD10 were partly explained by the very active neurogenic and gliogenic processes in late pregnancy [62]. However, these molecular recoveries did not exclude permanent subcellular abnormalities. A delay in cell production could cause abnormal connections or reduce cell integration into the network, and lead to abnormal behavioral phenotypes. Supporting this hypothesis, higher levels of non aligned cells (PnD140-160) [63] and a poor arborization of neurons (PnD60) [64] are reported in hippocampus of offspring prenatally exposed to LPS in late gestation. In addition, recent studies associated with mGluR5 and GABA receptors in different psychiatric disorders [65], propose a potent modulatory function of mGluR5 in the excitatory/inhibitory balance, suggesting that network activity could be modulated by mGluR5 activity.

Prenatal immune challenges are models of psychiatric diseases in which a decrease in performance of the brain network is suspected to be a key factor in the brain-related behavioral symptoms [54, 66]. Our study demonstrated a new downstream pathway of mGluR5 (PBR receptor), known to modulate of astrocyte functions, but defective in the offspring prenatally exposed to LPS. The disconnection of mGluR5-PBR pathway in astrocytes suggests a loss of sensitivity of the astrocytes to detect glutamate, the main excitatory neurotransmitter in brain. This desensitization might impair the modulation of synaptic activity by astrocytes [64]. The lack of this inter-cellular regulation might induce network failure, and consequently behavioral disorders.

### Relevance to humans

Recently there has been a growing interest for mGluR5 targeted therapeutic approaches in several central and peripheral diseases [64]. In schizophrenia, mGluR5 activators are known to reduce the negative and positive symptoms [3, 67]. Among the suspected mechanisms, potentiating of NMDA response by positive allosteric modulators (PAMs) [68] is suspected to improve cognitive deficits of this disease [67] while the antipsychotic effects of PAMs could be the consequence of the modulatory effect on the mesolimbic dopaminergic pathway [3], as suggested by the decrease in the basal dopamine levels in the nucleus accumbens induced by ADX47273 (mGluR5 PAM) [69]. In counterpart, many preclinical studies have reported an anxiolytic effect of mGluR5 negative modulator [70–72] and these observations were supported by a clinical study using fenobam (negative allosteric modulator of mGluR5) [73, 74]. Our studies demonstrate that PET imaging using [^18^F]FPEB and [^11^C]PBR28 is a new and non-invasive approach to evaluate *in vivo* how mGluR5 focused treatment can modulate PBR signaling in astrocyte. The developed methods are easily translatable for human studies especially since the used PET imaging ligands are already in human use.

### Methodologic limitations

The methodological limitation is that *in vivo* quantifications of PBR and mGluR5 are based on the affinity and specificity of the radioactive ligands to bind the receptor. This technical limit is valid for all quantifications based on an affinity approach, including the binding of ligand with its receptor or the ability of an antibody to recognize a molecular structure. [^18^F]FPEB is developed from the 2-methyl-6-(phenylethynyl)pyridine (MPEP) scaffold and binds the same site as MPEP in a fully competitive manner [75]. Many recent studies demonstrated that [^18^F]FPEB is currently a ligand of choice to target mGluR5. Receptor autoradiography studies in tissue sections have confirmed that the regional distribution of [^18^F]FPEB in mammalian central nervous system is consistent with that of mGluR5 [76]. All of these studies support [^18^F]FPEB as an acceptable ligand to image mGluR5 in the brain. Concerning PBR28, recent *in vivo* studies demonstrated that specific binding of [^11^C]PK11195, the most common radioactive ligand to image PBR over the past 10 years, was approximately 80-fold lower than that reported for [^11^C]PBR28 in monkey brain. Another important factor is the pharmacodynamics properties of the compound. To bind brain receptor, a molecule must cross the blood brain barrier. Since the brain permeability to [^18^F]FPEB and [^11^C]PBR28 was not investigated in normal mice and mice prenatally exposed to LPS, we cannot exclude pharmacodynamic change. In summary, the limitations of this study are not different from other studies.

### Major conclusions

- The expression of inflammatory marker TSPO/PBR is linked to the activation of mGluR5 in a mouse model of LPS prenatal immune challenge during maturation.
- The mGluR5 modulates the astrocyte functions and this pathway is defective in offspring prenatally exposed to LPS.
- The disconnection of mGluR5-PBR pathway in astrocytes suggests a loss of sensitivity of the astrocytes to detect glutamate, which might impair the neuronal-astrocyte inter-cellular network and consequently advance the development of behavioral disorders.

## Supporting Information

S1 Fig Effects of maturation and prenatal treatment on [^11^C]PBR binding potential in different brain structures. (TIF)

S2 Fig Effects of postnatal MTEP treatment on [^18^F]FPEB binding potential. (TIF)

S3 Fig Effects of postnatal MTEP treatment on [^11^C]PBR binding potential. (TIF)

S1 File (DOCX)

S2 File (DOCX)

S3 File (DOCX)

S1 Supplemental Discussion Hyperactivity of neuronal and glial mGluR5 is a potent pathway to reduce glutamate neurotoxicity and, subsequently, reduce astrocyte activation. (DOCX)

S1 Table Statistical values. (DOCX)
