# Supplementary figures and images for: Loss of Metabotropic Glutamate Receptor 5 Function on Peripheral Benzodiazepine Receptor in Mice Prenatally Exposed to LPS
Source: PLoS One. 2015 Nov 4;10(11):e0142093. doi: 10.1371/journal.pone.0142093 (PMC4633140; doi:10.1371/journal.pone.0142093)

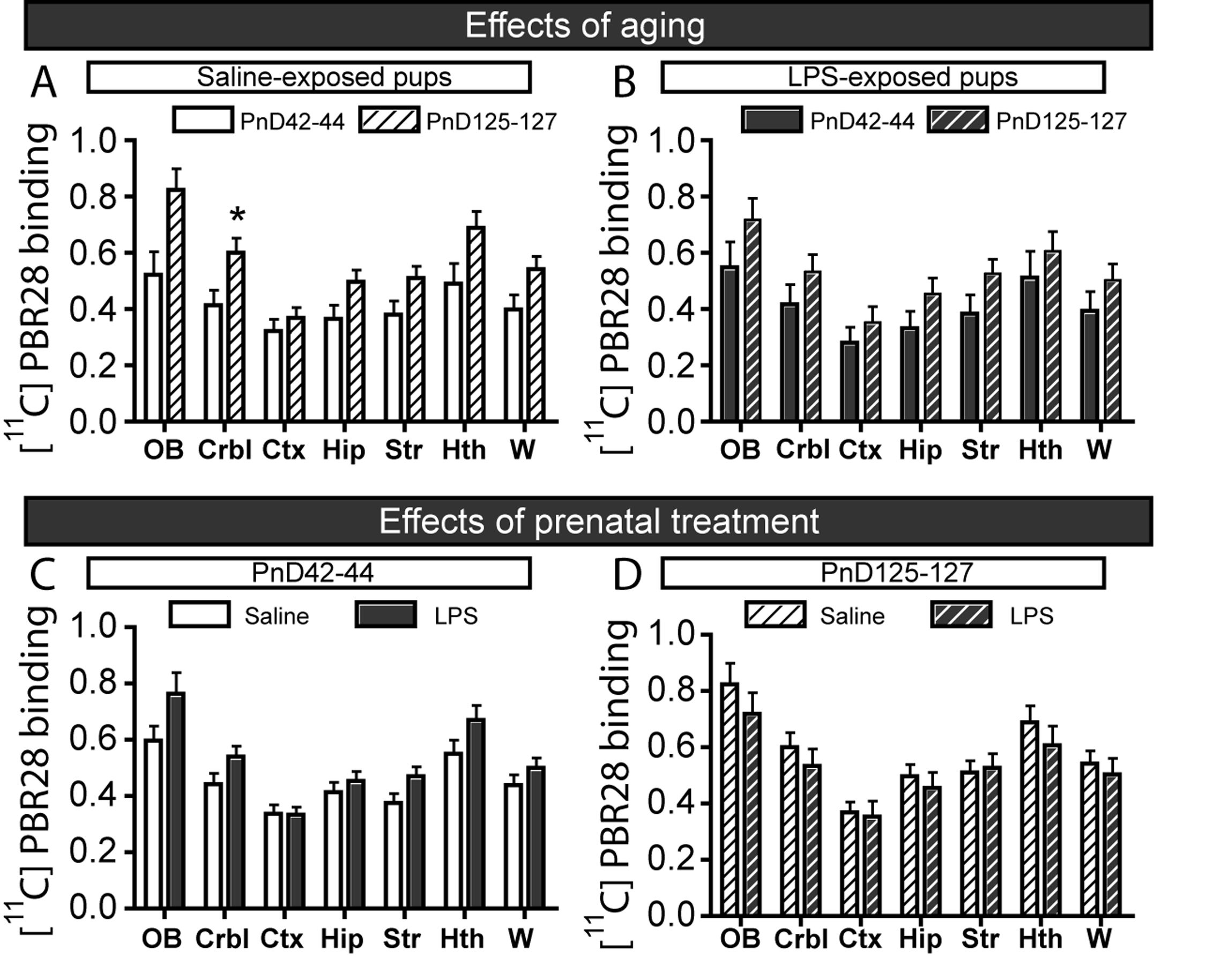

Supplement: S1 Fig — (TIF) [file pone.0142093.s001.tif]

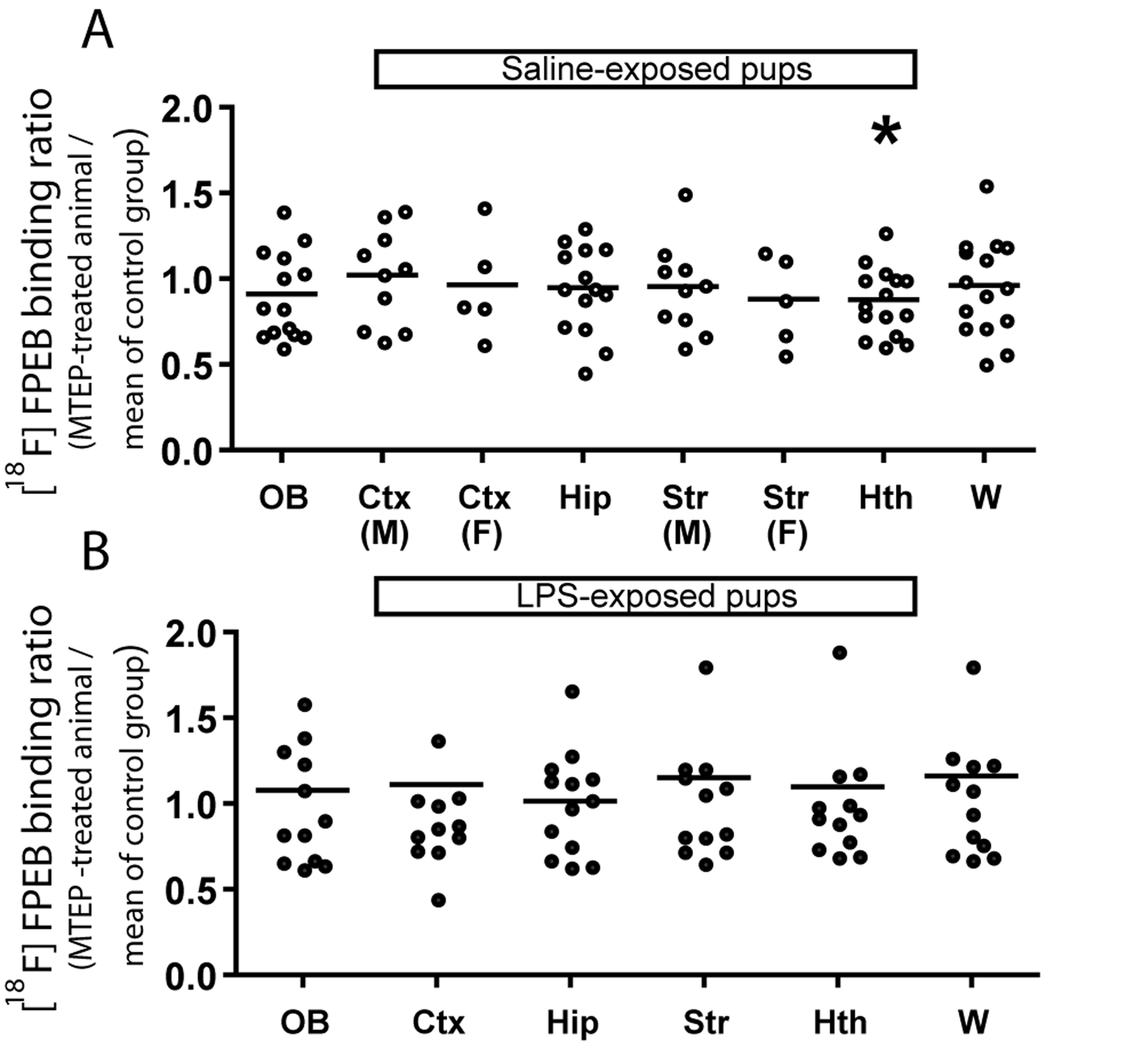

Supplement: S2 Fig — (TIF) [file pone.0142093.s002.tif]

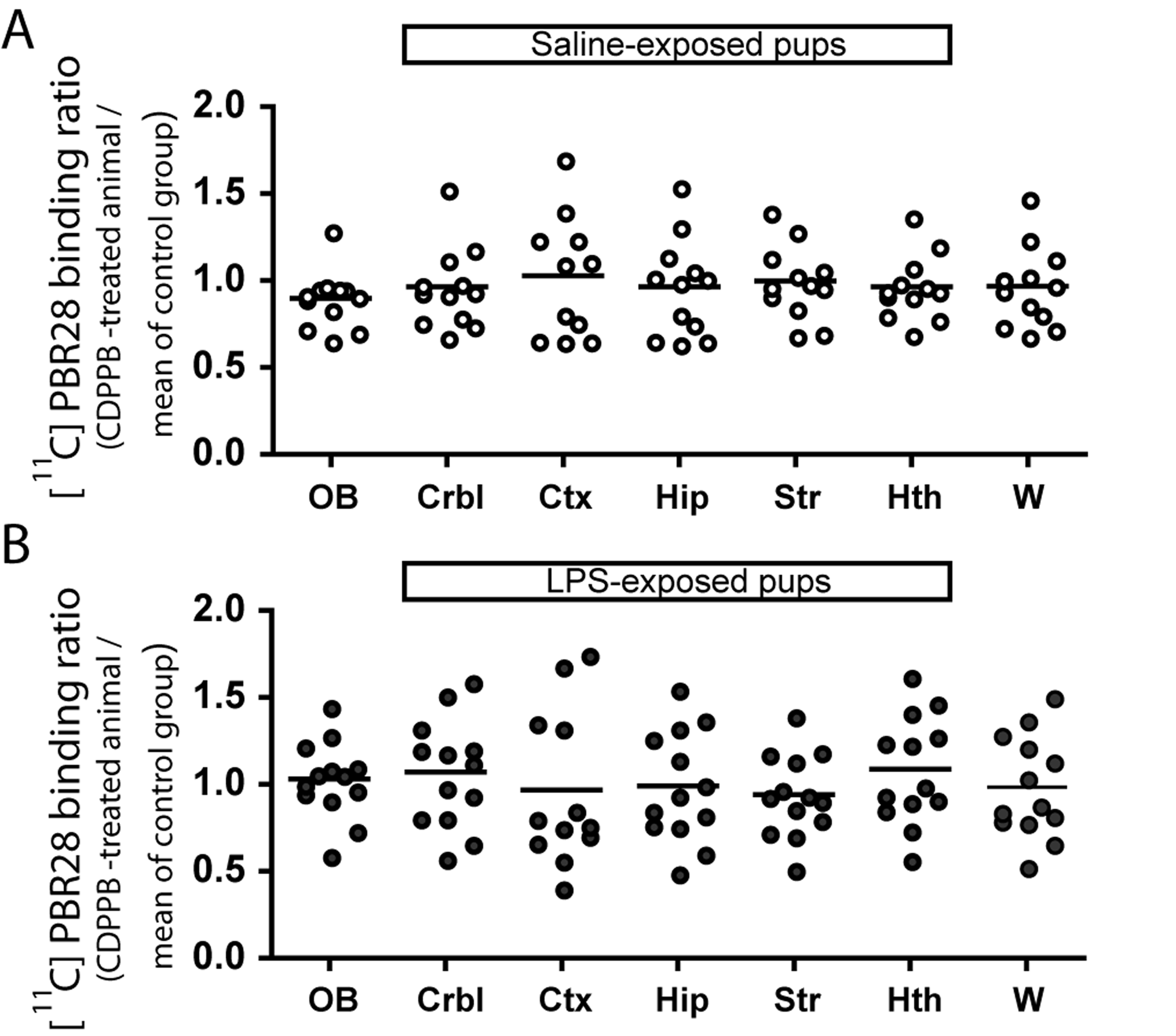

Supplement: S3 Fig — (TIF) [file pone.0142093.s003.tif]
