# Supplementary material for: Loss of Metabotropic Glutamate Receptor 5 Function on Peripheral Benzodiazepine Receptor in Mice Prenatally Exposed to LPS
Source: PLoS One. 2015 Nov 4;10(11):e0142093. doi: 10.1371/journal.pone.0142093 (PMC4633140; doi:10.1371/journal.pone.0142093)
Supplement: S1 File — (DOCX) [file pone.0142093.s004.docx]

**S1 Fig.** **Effects of maturation and prenatal treatment on [^11^C]PBR binding potential in different brain structures.**

[^11^C]PBR binding potential increased in the cerebellum of saline-treated offspring during maturation (A), whereas no significant change was observed in the offspring prenatally exposed to LPS (B). Saline- and LPS-exposed offspring showed similar level of [^11^C]PBR accumulation in the brain at PnD42-44 (C) and PnD125-127 (D). (E) Coronal slices of the hippocampal and striatal level of [^11^C]PBR in the offspring prenatally exposed to saline or LPS at PnD 42-44 and PnD 125-27. Values are expressed as mean ± SEM. Abbreviations: [^11^C] PBR28, peripheral benzodiazepine receptor 28; Ctx, cortex; Crbl, cerebellum; Hip, hippocampus; Hth, hypothalamus; OB, olfactory bulb; PnD, postnatal day; Str, striatum; W, whole brain. *p < 0.05, **p < 0.01. Statistical analyses were performed using paired t-test (OB, Crbl, Ctx, Hip, Str and W of panel A; OB, Crbl, Ctx, Str, Hth and W of panel B) or Wilcoxon matched pairs test (Hth of panel A; Hip of panel B). The number of animals was 12 (PnD42-44) and 8-9 (PnD125-127) for the saline-exposed offspring, whereas it was 8 (PnD42-44) and 10-11 (PnD125-127) for the LPS-exposed group. Unpaired Student’s t-test (Crbl, Hip, Str, Hth and W for panel C; all comparisons for panel D) and Mann-Whitney test (OB and Ctx for panel C) were used to compare the effects of the prenatal treatment. The number of animals was 28-29 (PnD42-44) and 8-9 (PnD125-127) for the saline-exposed offspring, whereas it was 27-29 (PnD42-44) and 10-11 (PnD125-127) for the LPS-exposed group.
