# Supplementary material for: Loss of Metabotropic Glutamate Receptor 5 Function on Peripheral Benzodiazepine Receptor in Mice Prenatally Exposed to LPS
Source: PLoS One. 2015 Nov 4;10(11):e0142093. doi: 10.1371/journal.pone.0142093 (PMC4633140; doi:10.1371/journal.pone.0142093)
Supplement: S2 File — (DOCX) [file pone.0142093.s005.docx]

**S2 Fig.**  **Effects of postnatal MTEP treatment on [^18^F]FPEB binding potential.**

PET imaging showed a lower level of [^18^F]FPEB accumulation in the hypothalamus of the mice prenatally exposed to saline following MTEP treatment (A). No effect of MTEP is observed in the LPS-exposed offspring (B). Values are expressed as mean ± SEM. Abbreviations: MTEP, 3-((2-methyl-4-thiazolyl)ethynyl)pyridine; [^18^F]FPEB, [^18^F]fluoro-5-(2-pyridinylethynyl) benzonitrile; Ctx, cortex; Hip, hippocampus; Hth, hypothalamus; OB, olfactory bulb; PnD, postnatal day; Str, striatum; W, whole brain. *p < 0.05. Statistical analyses were performed using one-sample t test (all comparisons of panel A; OB and Hip of panel B) or Wilcoxon signed-rank test (Ctx, Str, Hth and W of panel B). A gender difference was observed in the cortex and striatum of the mice prenatally exposed to saline (Panel A). Therefore, the sexes were analyzed separately. The number of animals was 14 - 15 for saline group (except when the sexes were analyzed separately), whereas it was 13 for LPS group.
