# Supplementary material for: Loss of Metabotropic Glutamate Receptor 5 Function on Peripheral Benzodiazepine Receptor in Mice Prenatally Exposed to LPS
Source: PLoS One. 2015 Nov 4;10(11):e0142093. doi: 10.1371/journal.pone.0142093 (PMC4633140; doi:10.1371/journal.pone.0142093)
Supplement: S3 File — (DOCX) [file pone.0142093.s006.docx]

**S3 Fig.** **Effects of postnatal MTEP treatment on [^11^C]PBR binding potential.**

Postnatal MTEP treatment did not change [^11^C]PBR binding potential in the quantified brain regions of the saline- (A) or the LPS-exposed (B) offspring. Values are expressed as mean ± SEM. Abbreviations: MTEP, 3-((2-methyl-4-thiazolyl)ethynyl)pyridine; [^11^C] PBR28, peripheral benzodiazepine receptor 28; Ctx, cortex; Crbl, cerebellum; Hip, hippocampus; Hth, hypothalamus; OB, olfactory bulb; PnD, postnatal day; Str, striatum; W, whole brain. *p < 0.05, **p < 0.01. Statistical analyses were performed using one-sample t test (Crbl Ctx, Hip, Str, Hth and W of panel A; OB, Crbl, Hip, Str, Hth and W of panel B) or Wilcoxon signed-rank test (OB of panel A; Ctx of panel B). The number of animals was 11-12 for the saline group and 12-13 for the LPS group.
