# Supplementary material for: Loss of Metabotropic Glutamate Receptor 5 Function on Peripheral Benzodiazepine Receptor in Mice Prenatally Exposed to LPS
Source: PLoS One. 2015 Nov 4;10(11):e0142093. doi: 10.1371/journal.pone.0142093 (PMC4633140; doi:10.1371/journal.pone.0142093)
Supplement: S1 Supplemental Discussion — (DOCX) [file pone.0142093.s007.docx]

**S1. Supplemental Discussion**

**Hyperactivity of neuronal and glial mGluR5 is a potent pathway to reduce glutamate neurotoxicity and astrocyte activation**

The inflammatory processes reported in the fetal brain following an exposure to LPS in late gestation [1-3] are proposed to be key factors in the enhanced sensitivity to excitotoxic lesions in newborn rats [4], suggesting that the brain inflammation interferes with neurodevelopmental processes during the gestational period and conducts to a suboptimal brain network. The mechanisms underlying this hypersensitivity to excitatory processes are not clear. The role of astrocytes in glutamate homeostasis [5, 6] and the expression of mGluR5 in astrocytes, activated by inflammatory stimulus [7] during epileptic seizures [8], support the idea that astrocytic mGluR5 contributes to higher sensitivity of excitotoxicity in LPS-exposed newborn. The studies showing that mGluR5 activation inhibits the inflammatory-dependent neurotoxicity in glial cell culture [9-11] and reduces the number of reactive glial cells in some neuropathological diseases like ischemia and spinal cord injury [9, 12] suggest neuroprotective effets of glial mGluR5.

The neuronal mGluR5 is involved also in glutamate homeostasis and has the key role in the protection of brain to glutamate-related neurotoxicity. mGluR5 activation in neuron drives the NMDA receptor, NR2B to NR2A subunit switch at synaptic membrane [13]. NR2B-containing NMDARs exhibit slower kinetics than NR2A-containing receptors [14, 15], changing the NMDAR function [16] and altering the amount of calcium influx through the pore [17]. NR2B to NR2A subunit switch at synaptic membrane is known to reduce NMDA-related neurotoxicity in the cell culture of PC12 (neuroblasts) [18] and is a potential mechanism to prevent excitotoxicity mediated by activated astrocytes [19]. In addition, excessive activity of neuronal mGluR5 increases the internalization of AMPA receptors and causes spontaneous action potential-driven network activity without synaptic stimulation by an exogenous agonist in model of Fragile X mental retardation [20]. In summary, the effects of neuronal mGluR5 on NMDA and AMPA receptors result in a reduction of postsynaptic responses to glutamate. The loss of synaptic efficiency is a potent mechanism to prevent excitotoxicity, a function for which our model seems to be particularly sensitive [4]. In addition, the loss of postsynaptic responses may regulate the phenotype and the function of astrocytes, because the glutamate released during neuronal activity modulates calcium entry to astrocytes by a mechanism, which is dependent on the group I mGluRs [21]. The intracellular calcium is the key factor in astrocyte activation [22]. A reduction of synaptic strength is a potent mechanism by which neuronal mGluR5 may regulate the level of reactive astrocyte and, consequently, the level of PBR [23, 24] and GFAP [24, 25]. Therefore, a functional change of mGluR5 in neuron was the potent mechanism in our results. To conclude, mGluR5 in both neurons and astrocytes was able to modulate astrocytic function and downregulate expression of PBR/GFAP. Consequently, our data was not able to discriminate which receptor (neuronal or astrocytic) was involved in the the regulation of GFAP/PBR by mGluR5 and which pathways were dysfunctional in the offspring prenatally exposed to LPS.

**Supplemental Bibliography**

1. Arsenault D, St-Amour I, Cisbani G, Rousseau LS, Cicchetti F (2013) The different effects of LPS and poly I:C prenatal immune challenges on the behavior, development and inflammatory responses in pregnant mice and their offspring. Brain Behav Immun 38: 77-90.

2. Golan H, Stilman M, Lev V, Huleihel M (2006) Normal aging of offspring mice of mothers with induced inflammation during pregnancy. Neuroscience 141: 1909-1918.

3. Golan HM, Lev V, Hallak M, Sorokin Y, Huleihel M (2005) Specific neurodevelopmental damage in mice offspring following maternal inflammation during pregnancy. Neuropharmacol 48: 903-917.

4. Rousset C, Gressens P, Binet A, Andres C, Chalon S, et al. (2005) 318 Maternal LPS Enhances Excitotoxic Brain Lesions in Newborn Rats. Pediatric Research 58: 409.

5. Schousboe A, Waagepetersen HS (2005) Role of astrocytes in glutamate homeostasis: implications for excitotoxicity. Neurotox Res 8: 221-225.

6. Schousboe A, Westergaard N, Sonnewald U, Petersen SB, Yu AC, et al. (1992) Regulatory role of astrocytes for neuronal biosynthesis and homeostasis of glutamate and GABA. Prog Brain Res 94: 199-211.

7. Berger JV, Dumont AO, Focant MC, Vergouts M, Sternotte A, et al. (2012) Opposite regulation of metabotropic glutamate receptor 3 and metabotropic glutamate receptor 5 by inflammatory stimuli in cultured microglia and astrocytes. Neuroscience 205: 29-38.

8. Aronica E, van Vliet EA, Mayboroda OA, Troost D, da Silva FH, et al. (2000) Upregulation of metabotropic glutamate receptor subtype mGluR3 and mGluR5 in reactive astrocytes in a rat model of mesial temporal lobe epilepsy. Eur J Neurosci 12: 2333-2344.

9. Byrnes KR, Loane DJ, Stoica BA, Zhang J, Faden AI (2012) Delayed mGluR5 activation limits neuroinflammation and neurodegeneration after traumatic brain injury. J Neuroinflammation 9: 43.

10. Byrnes KR, Stoica B, Loane DJ, Riccio A, Davis MI, et al. (2009) Metabotropic glutamate receptor 5 activation inhibits microglial associated inflammation and neurotoxicity. Glia 57: 550-560.

11. Loane DJ, Stoica BA, Pajoohesh-Ganji A, Byrnes KR, Faden AI (2009) Activation of metabotropic glutamate receptor 5 modulates microglial reactivity and neurotoxicity by inhibiting NADPH oxidase. J Biol Chem 284: 15629-15639.

12. Byrnes KR, Stoica B, Riccio A, Pajoohesh-Ganji A, Loane DJ, et al. (2009) Activation of metabotropic glutamate receptor 5 improves recovery after spinal cord injury in rodents. Ann Neurol 66: 63-74.

13. Matta JA, Ashby MC, Sanz-Clemente A, Roche KW, Isaac JT (2011) mGluR5 and NMDA receptors drive the experience- and activity-dependent NMDA receptor NR2B to NR2A subunit switch. Neuron 70: 339-351.

14. Williams K (1993) Ifenprodil discriminates subtypes of the N-methyl-D-aspartate receptor: selectivity and mechanisms at recombinant heteromeric receptors. Mol Pharmacol 44: 851-859.

15. Arsenault D, Zhang ZW (2006) Developmental remodelling of the lemniscal synapse in the ventral basal thalamus of the mouse. J Physiol 573: 121-132.

16. Erreger K, Dravid SM, Banke TG, Wyllie DJ, Traynelis SF (2005) Subunit-specific gating controls rat NR1/NR2A and NR1/NR2B NMDA channel kinetics and synaptic signalling profiles. J Physiol 563: 345-358.

17. Bloodgood BL, Sabatini BL (2009) NMDA Receptor-Mediated Calcium Transients in Dendritic Spines.

18. Dai SH, Qin N, Chen T, Luo P, Zhang L, et al. (2014) Activation of mGluR5 attenuates NMDA-induced neurotoxicity through disruption of the NMDAR-PSD-95 complex and preservation of mitochondrial function in differentiated PC12 cells. Int J Mol Sci 15: 10892-10907.

19. Ahlemeyer B, Kolker S, Zhu Y, Hoffmann GF, Krieglstein J (2002) Increase in glutamate-induced neurotoxicity by activated astrocytes involves stimulation of protein kinase C. J Neurochem 82: 504-515.

20. Nakamoto M, Nalavadi V, Epstein MP, Narayanan U, Bassell GJ, et al. (2007) Fragile X mental retardation protein deficiency leads to excessive mGluR5-dependent internalization of AMPA receptors. Proc Natl Acad Sci U S A 104: 15537-15542.

21. Wallach G, Lallouette J, Herzog N, De Pitta M, Ben Jacob E, et al. (2014) Glutamate mediated astrocytic filtering of neuronal activity. PLoS Comput Biol 10: e1003964.

22. Kanemaru K, Kubota J, Sekiya H, Hirose K, Okubo Y, et al. (2013) Calcium-dependent N-cadherin up-regulation mediates reactive astrogliosis and neuroprotection after brain injury. Proc Natl Acad Sci U S A 110: 11612-11617.

23. Guilarte TR, Kuhlmann AC, O'Callaghan JP, Miceli RC (1995) Enhanced expression of peripheral benzodiazepine receptors in trimethyltin-exposed rat brain: a biomarker of neurotoxicity. Neurotoxicology 16: 441-450.

24. Kuhlmann AC, Guilarte TR (2000) Cellular and subcellular localization of peripheral benzodiazepine receptors after trimethyltin neurotoxicity. J Neurochem 74: 1694-1704.

25. Eng LF, Ghirnikar RS (1994) GFAP and astrogliosis. Brain Pathol 4: 229-237.
