# Supplementary material for: Loss of Metabotropic Glutamate Receptor 5 Function on Peripheral Benzodiazepine Receptor in Mice Prenatally Exposed to LPS
Source: PLoS One. 2015 Nov 4;10(11):e0142093. doi: 10.1371/journal.pone.0142093 (PMC4633140; doi:10.1371/journal.pone.0142093)
Supplement: S1 Table — (DOCX) [file pone.0142093.s008.docx]

**S1 Table.** **Statistical values.** The used statistical methods and obtained p- values in each treatment group and brain region.

|  | **Statistics** | **U, T, F or W value / p value** | **Statistics** | **U, T, F or W value/ p value** |
| --- | --- | --- | --- | --- |
| **S1 Fig. (PBR binding potential)** | | | | |
| Brain | **panel A** | | **panel B** | |
| region | *Saline-exposed offspring (PnD42-44 vs PnD125-127)* | | *LPS-exposed offspring (PnD42-44 vs PnD125-127)* | |
| OB | Paired T-test | T (7) = 2.199 / p = 0.064 | Paired T-test | T (7) = 1.402 / p = 0.204 |
| Crbl | Paired T-test | T (7) = 2.389 / **p = 0.048** | Paired T-test | T (7) = 1.458 / p = 0.188 |
| Ctx | Paired T-test | T (6) = 0.821 / p = 0.445 | Paired T-test | T (6) = 0.967 / p = 0.371 |
| Hip | Paired T-test | T (7) = 1.825 / p = 0.111 | Wilcoxon matched-pairs signed rank test | W (7.00, -29.00) = -22 / p = 0.148 |
| Str | Paired T-test | T (7) = 1.788 / p = 0.117 | Paired T-test | T (7) = 1.694 / p = 0.134 |
| Hth | Wilcoxon matched-pairs signed rank test | W (7.00, -29.00) = -22 / p = 0.148 | Paired T-test | T (6) = 0.761 / p = 0.476 |
| W | Paired T-test | T (7) = 1.994 / p = 0.086 | Paired T-test | T (7) = 1.435 / p = 0.195 |
| Brain | **panel C** | | **panel D** | |
| region | *PnD42-44 (saline vs LPS prenatal exposition)* | | *PnD125-127 (saline vs LPS prenatal exposition)* | |
| OB | Mann-Whitney Test | U (747, 849) = 312.0 / p = 0.195 | Unpaired T-test | T (18) = 0.943 / p = 0.358 |
| Crbl | Unpaired T-test | T (55) = 1.757 / p = 0.084 | Unpaired T-test | T (18) = 0.813 / p = 0.427 |
| Ctx | Unpaired T-test | T (56) = 0.096 / p = 0.924 | Unpaired T-test | T (16) = 0.214 / p = 0.833 |
| Hip | Unpaired T-test | T (56) = 0.770 / p = 0.445 | Unpaired T-test | T (18) = 0.570 / p = 0.576 |
| Str | Mann-Whitney Test | U (590, 841) = 265.0 / p = 0.132 | Unpaired T-test | T (18) = 0.253 / p = 0.803 |
| Hth | Unpaired T-test | T (56) = 1.664 / p = 0.102 | Unpaired T-test | T (17) = 0.888 / p = 0.387 |
| W | Unpaired T-test | T (56) = 1.296 / p = 0.247 | Unpaired T-test | T (18) = 0.510 / p = 0.617 |
| **S2 Fig. (FPEB binding potential)** | | | | |
| Brain | **panel A** | | **panel B** | |
| region | *Effect of MTEP in saline-exposed offspring (PnD117-119)* | | *Effect of MTEP in LPS-exposed offspring (PnD117-119)* | |
| OB | One sample t test | T (13) = 1.321 / p = 0.209 | One sample t test | T (12) = 0.604 / p = 0.557 |
| Crbl | ------ | ------ | ------ | ------ |
| Ctx | (M) One sample t test | T (9) = 0.246 / p = 0.811 |  |  |
| Ctx | (F) One sample t test | T (4) = 0.253 / p = 0.813 | Wilcoxon signed rank test | W (40.00, -51.00) = -11.00 /p = 0.735 |
| Hip | One sample t test | T (13) = 0.768 / p = 0.457 | One sample t test | T (12) = 0.161 / p = 0.875 |
| Str | (M) One sample t test | T (9) = 0.556 / p = 0.592 |  |  |
| Str | (F) One sample t test | T (4) = 1.009 / p = 0.370 | Wilcoxon signed rank test | W (46.00, -45.00) = 1.00 / p = 1.000 |
| Hth | One sample t test | T (14) = 2.407 / **p = 0.031** | Wilcoxon signed rank test | W (39.00, -42.00) = -13.00 /p = 0.685 |
| W | One sample t test | T (14) = 0.522 / p = 0.610 | Wilcoxon signed rank test | W (50.00, -41.00) = 9.00 / p = 0.787 |
| **S3 Fig. (PBR binding potential)** | | | | |
| Brain | **panel A** | | **panel B** | |
| region | *Effect of MTEP in saline-exposed offspring (PnD125-127)* | | *Effect of MTEP in LPS-exposed offspring (PnD117-119)* | |
| OB | One sample t test | T (11) = 2.113 / p = 0.058 | One sample t test | T (12) = 0.531 / p = 0.605 |
| Crbl | One sample t test | T (11) = 0.543 / p = 0.598 | One sample t test | T (12) = 0.816 / p = 0.430 |
| Ctx | One sample t test | T (10) = 0.277 / p = 0.787 | Wilcoxon signed rank test | W (37.00, -41.00) = -4.00 / p = 0.909 |
| Hip | One sample t test | T (11) = 0.418 / p = 0.684 | One sample t test | T (12) = 0.089 / p = 0.930 |
| Str | One sample t test | T (11) = 0.053 / p = 0.959 | One sample t test | T (12) = 0.881 / p = 0.395 |
| Hth | One sample t test | T (11) = 0.650 / p = 0.529 | One sample t test | T (12) = 1.041 / p = 0.318 |
| W | One sample t test | T (11) = 0.481 / p = 0.640 | One sample t test | T (12) = 0.126 / p = 0.902 |
